# Levels of DNA Methylation Vary at CpG Sites across the *BRCA1* Promoter, and Differ According to Triple Negative and “*BRCA*-Like” Status, in Both Blood and Tumour DNA

**DOI:** 10.1371/journal.pone.0160174

**Published:** 2016-07-27

**Authors:** Sarah L. Daniels, George J. Burghel, Philip Chambers, Shadi Al-Baba, Daniel D. Connley, Ian W. Brock, Helen E. Cramp, Olena Dotsenko, Octavia Wilks, Lynda Wyld, Simon S. Cross, Angela Cox

**Affiliations:** 1 Academic Unit of Molecular Oncology, Department of Oncology and Metabolism, University of Sheffield, Sheffield Medical School, Beech Hill Road, Sheffield, S10 2RX, United Kingdom; 2 Leeds Institute of Cancer and Pathology, Section of Pathology and Tumour Biology, Wellcome Trust Brenner Building, University of Leeds, St. James’s University Hospital, Beckett Street, Leeds, LS9 7TF, United Kingdom; 3 Sheffield Diagnostic Genetics Service, Sheffield Children’s NHS Foundation Trust, Western Bank, Sheffield, S10 2TH, United Kingdom; 4 Department of Histopathology, Royal Hallamshire Hospital, Sheffield Teaching Hospitals NHS Foundation Trust, Glossop Road, Sheffield, S10 2JF, United Kingdom; 5 Academic Unit of Pathology, Department of Neuroscience, Faculty of Medicine, Dentistry & Health, The University of Sheffield, Beech Hill Road, Sheffield, S10 2RX, United Kingdom; 6 Academic Unit of Surgical Oncology, University of Sheffield Medical School, Beech Hill Road, Sheffield, S10 2RX, United Kingdom; 7 Sheffield Institute for Nucleic Acids (SInFoNiA), University of Sheffield, Western Bank, Sheffield, S10 2TN, United Kingdom; King Faisal Specialist Hospital and Research Center, SAUDI ARABIA

## Abstract

Triple negative breast cancer is typically an aggressive and difficult to treat subtype. It is often associated with loss of function of the *BRCA1* gene, either through mutation, loss of heterozygosity or methylation. This study aimed to measure methylation of the *BRCA1* gene promoter at individual CpG sites in blood, tumour and normal breast tissue, to assess whether levels were correlated between different tissues, and with triple negative receptor status, histopathological scoring for BRCA-like features and BRCA1 protein expression. Blood DNA methylation levels were significantly correlated with tumour methylation at 9 of 11 CpG sites examined (p<0.0007). The levels of tumour DNA methylation were significantly higher in triple negative tumours, and in tumours with high BRCA-like histopathological scores (10 of 11 CpG sites; p<0.01 and p<0.007 respectively). Similar results were observed in blood DNA (6 of 11 CpG sites; p<0.03 and 7 of 11 CpG sites; p<0.02 respectively). This study provides insight into the pattern of CpG methylation across the *BRCA1* promoter, and supports previous studies suggesting that tumours with *BRCA1* promoter methylation have similar features to those with *BRCA1* mutations, and therefore may be suitable for the same targeted therapies.

## Introduction

The triple negative (TN) subtype of breast cancer accounts for 10–17% of all breast carcinomas [1–4]. Triple negative tumours are more likely to be of higher grade, to present with nodal or distant metastases, and there is a relative lack of effective therapies compared to other cancer subtypes, which all contribute to poor disease-free and overall survival [5]. By definition, these tumours are oestrogen receptor (ER) and progesterone receptor (PR) negative and negative for human epidermal growth factor receptor (*HER2*). Triple negative tumours are known to be a heterogeneous group with a significant proportion displaying the basal-like phenotype; with overexpression of cytokeratin 5/6(CK) and epidermal growth factor receptor (EGFR) proteins. However, all other molecular subtypes of breast cancer are also present in TN cohorts [6, 7]. Recent comprehensive RNA and DNA profiling analyses have identified at least four distinct subtypes of triple negative breast cancers that may have specific therapeutic targets based on their molecular signatures [8, 9].

The Breast Cancer susceptibility gene 1 (*BRCA1*) is the most commonly mutated gene in familial breast cancer cases and is strongly associated with both the TN subtype and basal-like breast tumours [10, 11]. Over 50% of *BRCA1* mutation-associated tumours are TN [12], however, *BRCA1* mutations are rarely found in sporadic breast cancer cases and less than 15% of TN tumours harbour *BRCA1* mutations [13–15]. The *BRCA1* gene is involved in homologous recombination DNA repair, which is the least error-prone mechanism for cells to repair double-stranded DNA breaks [16]. Cells that lack functional *BRCA1*, whether it is through mutation, loss of heterozygosity or epigenetic mechanisms, are deficient in homologous recombination repair. These cells utilise alternative DNA repair mechanisms that are more error prone, resulting in tumours with high levels of genomic instability [17, 18], a high frequency of TP53 mutations [19] and numerous copy number aberrations [20]. These characteristic patterns of gains and losses of genomic DNA associated with *BRCA1* mutant tumours can be used to identify a larger group of sporadic cancers that are molecularly similar but lack *BRCA1* mutations, known as BRCA1-like [20–22]. The term ‘BRCA-ness’ similarly refers to tumours in which no germline *BRCA1* mutation has been identified but which share histopathological features frequently found in *BRCA1* mutated tumours, including a high mitotic index, pushing borders, syncytial and circumscribed growth patterns [23, 24].

There is considerable evidence that epigenetic mechanisms, in particular hypermethylation of tumour suppressor gene promoters, represent an alternative method of gene silencing/ inactivation [7, 24–26]. Methylation of the *BRCA1* promoter in breast tumours is associated with a poor overall survival and disease-free survival and has been suggested as a biomarker to guide prognosis and targeted therapies [27–30]. Severson et al found that germline mutation and *BRCA1* promoter methylation overlap with BRCA1-like status (determined by copy number aberrations) in 70% and 79% of their samples respectively [20]. Triple negative tumours in young women with multiple BRCA1-like morphological features are associated with hypermethylation of the *BRCA1* promoter in blood DNA [23]. However, there remains debate regarding whether blood and tumour data are concordant for gene specific methylation [31]. Tumour *BRCA1* promoter methylation has been reported to predict response to platinum based chemotherapy agents and Poly(ADP-ribose) polymerase (PARP) inhibitors, therefore methylation status could potentially influence treatment decisions [32].

In order to examine the relationship between *BRCA1* promoter methylation, BRCA1 protein expression, triple negativity and BRCA1 associated histopathological features we have analysed blood samples from 658 women with sporadic breast cancer and 170 matched tumour samples; 71 (11%) and 35 (21%) of these samples were classified as TN respectively.

## Methods

### Study population and data collection

The study population comprised women diagnosed with invasive breast cancer at Sheffield Teaching Hospitals NHS Foundation Trust, UK, recruited as part of the Sheffield Breast Cancer Study. The study was approved by the Sheffield Research Ethics Committee, and all women provided written informed consent. Women were recruited in two cohorts between 1998–2008 and 2009–2014. Women in the earlier cohort were recruited from surgical outpatient clinics, whilst women in the later cohort were newly diagnosed and recruited at pre-operative assessment. Women with known *BRCA1*/2 gene mutations were excluded. Data on tumour grade, receptor status, nodal status and age at diagnosis were obtained from clinical notes, and menopausal status and family history of breast cancer were obtained from the patient by questionnaire administered by a research nurse. Samples from women with triple negative tumours were preferentially selected for *BRCA1* promoter methylation analysis.

### Sample collection and DNA isolation

Genomic DNA was extracted from peripheral blood mononuclear cells isolated from 6ml whole blood or 2ml ‘buffy coat’ samples according to the manufacturer’s protocol (Flexigene DNA extraction kit, Qiagen). The concentration of extracted DNA was quantified using a NanoDrop spectrophotometer (Thermo Scientific, ND-1000 software). DNA samples were stored at -80°C until required. Tumour or normal tissue DNA was isolated following macrodissection from five 10 micron paraffin sections per FFPE block (to ensure greater than 80% tumour cells), and extracted according to the manufacturer’s protocol (QIAamp DNA FFPE kit, Qiagen). Tumour DNA was eluted in a final volume of 70μl buffer and then quantified using a NanoDrop Spectrophotometer.

### Receptor status

For the 1998–2008 cohort, tumour receptor status for ER, PR and HER2 were determined by immunohistochemistry of triplicate tumour cores in tissue microarrays, and scored by SSC. Antibodies were as follows; ER: Vector 6F11/2 (1:50), PR: Vector 1A6 (1:40), HER2: Dako HercepTest Kit (pre-diluted). For the 2009–2014 cohort, ER and HER2 status determined according to UK guidelines were obtained from NHS histopathology records.

### Morphological scoring

Haematoxylin and Eosin (H&E) staining was performed on one slide per tumour and the slides were systematically reviewed by a consultant histopathologist (SSC), who scored them for the presence of nine BRCA1-associated morphological features; high mitotic index, malignant nuclear grade, little or no (<10%) tubule formation, trabecular growth pattern, syncytial growth pattern, pushing margins (>50%), circumscribed growth pattern, necrosis, moderate or intense lymphocytic infiltrate [23, 33].

### BRCA1 protein expression

BRCA1 immunohistochemistry was carried out using the anti-BRCA1 (Ab-1) mouse antibody (MS110 OP92 Calbiochem) on 5micron FFPE sections at 1:400 dilution as described previously [36]. MCF-7 cell line cytospins were used to provide positive and negative (no primary antibody) controls. Slides were scored for BRCA1 nuclear staining by SSC and OW. The percentage of positive nuclei were scored between 0 and 5, the intensity of nuclear staining was scored between 0 and 3, then these were added to form the combined score (Allred quick score). Tumours with a score equal or less than 4 were deemed to be negative for BRCA1 expression.

### Methylation analysis

**Fig 1:**
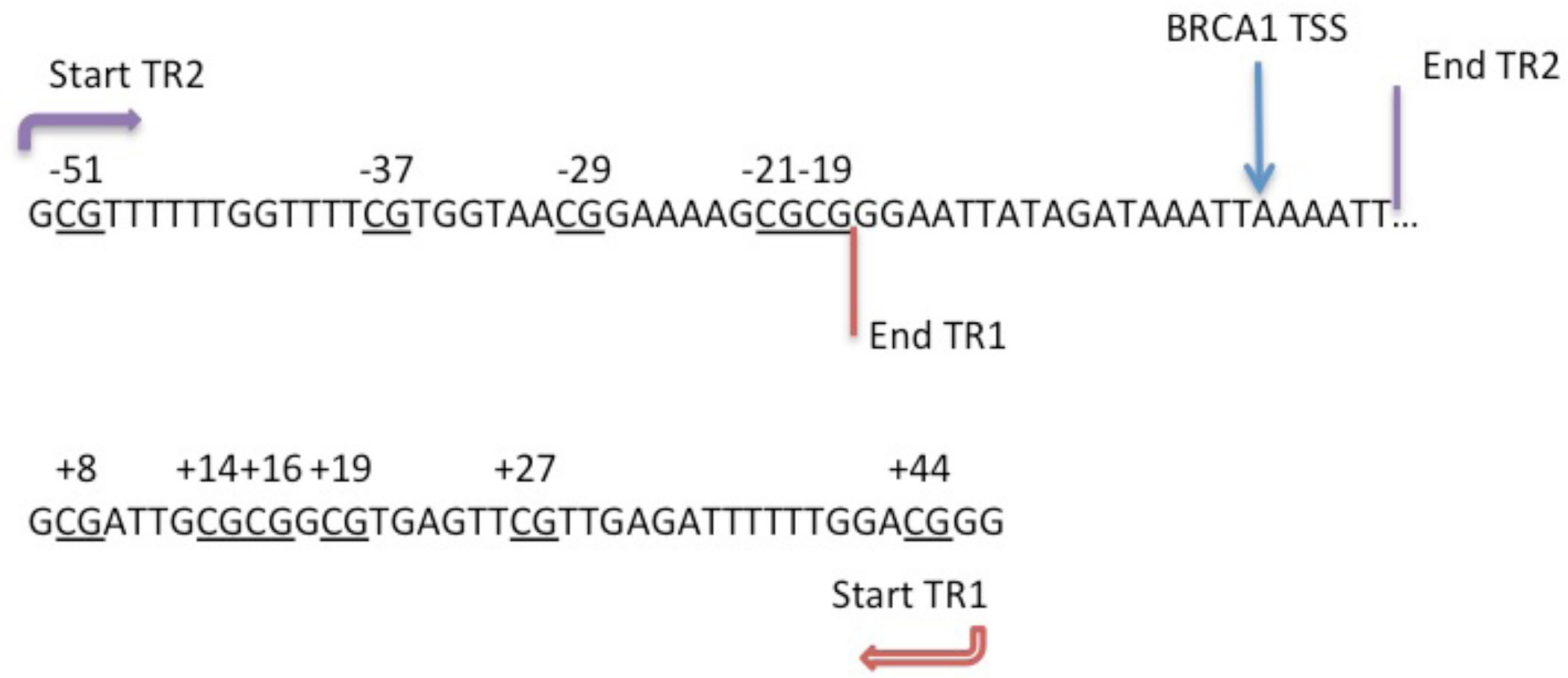
Diagram of the 11 CpG sites analysed by pyrosequencing. Individual CpG sites are underlined and numbering is relative to the *BRCA1* Transcription Start Site (TSS). The start and end of the two overlapping target regions are detailed. No CpGs were present in the overlapping section.

Sodium-bisulphite modification of blood and tumour DNA was performed using the CpGenome DNA modification kit (EMD Millipore, USA) according to the manufacturer’s protocol, to convert unmethylated cytosine residues to uracil. Sodium bisulphite-treated DNA was then analysed by pyrosequencing as described previously [34]. Two sets of pyrosequencing primers were designed for a 313 base pair (bp) region of the *BRCA1* promoter using Pyromark assay design software (version 2.0) and are detailed in Table 1. The pyrosequencing targets contained 11 CpG sites, which included all of those studied by Wong et al plus two additional adjacent sites (Fig 1) [23, 35]. CpG sites are referred to by their base pair position relative to the BRCA1 transcription start site (position zero). The oligonucleotides (Sigma Aldrich, Ebersberg, Germany) were reconstituted with deionised water at a stock concentration of 100pmol/μl.

**Table 1:** Details the primer sequences used for pyrosequencing.

		5’3’ sequence	Strand
**Target region 1**	F	(Biotin)TGATTTAGTATTTTGAGAGGTTGTTGTT	Sense
	R	CAATTATCTAAAAAACCCCACAACCTA	Reverse
	S	CCCACAACCTATCCC	Reverse
**Target region 2**	F	GTATTTTTGAGAGGTTGTTGTT	Sense
	R	(Biotin)AAACCCCACAACCTATCC	Reverse
	S	TTTGAGAGGTTGTTGTTTA	Sense

Primer orientation: Forward (F), Reverse (R), Sequencing (S).

PCR was performed using Hotstart taq DNA polymerase (Hotstart PCR kit, Qiagen) under the following PCR conditions; denaturation at 95°C for 15 minutes followed by 50 cycles of the following profile; 95°C for 20 seconds, 61°C for 20 seconds, 72°C for 20 seconds followed by a final 5 minutes extension at 72°C. The PCR products were analysed by gel electrophoresis on a 1.5% Agarose gel stained with ethidium bromide and visualised by UV trans-illumination prior to pyrosequencing. The 11 CpG sites were analysed by pyrosequencing using PyroMark Q96 MD and Pyromark Gold reagents (Qiagen AG, Basel, Switzerland). Bisulphite-modified universally methylated DNA (Chemicon International, NY) and distilled water were included in each run as positive and negative controls.

### Statistical Analysis

Correlation of methylation levels at individual CpG sites between matched tumour and blood DNA, and between matched pairs of tumour samples was based on Spearman’s rank correlation coefficient. Methylation levels between matched pairs of samples were compared using the Wilcoxon matched-pairs signed-ranks test. Unmatched groups were compared using the Mann-Whitney test. Associations between different pathological features were assessed using contingency tables. The data set used for these analyses is provided in S1 Table. All analyses were implemented in Stata V12.1 and all statistical tests were two-sided.

## Results

### Baseline characteristics of cases and controls

Blood methylation analysis was successfully carried out on 658 cases, of whom 170 had sufficient tumour tissue available for methylation analysis; 71 (11%) and 35 (21%) of these were classified as TN respectively. Normal breast tissue was available for 26 cases, and for 20 cases two tumour FFPE blocks were available from the same tumour. There were no significant differences in baseline demographics (age, menopausal status and history of first degree relative affected with breast cancer) between those from whom tumours were available and those where only blood was available (Table 2). However, the cases where tumours were available were of higher grade (p<0.001), more likely lymph node positive (p = 0.03) and more likely to be TN (p<0.001) (Table 2), reflecting the fact that cases with TN disease were preferentially selected for tumour analysis, and that tumours with sufficient tissue available for DNA extraction tend to be of higher grade and node positive since these features are associated with larger tumours.

**Table 2:** Study population demographics according to blood and tumour tissue availability.

	All cases with blood	Cases with blood only	Cases with blood and tumour	Blood plus tumour versus blood only	Cases with two tumour FFPE blocks	Cases with normal tissue
**Number, n**	658	488	170		20	26
**Median age at diag (range)**	60 (23–92)	60 (23–92)	58 (24–85)	p = 0.52	62.5 (24–84)	58.5 (39–84)
**First degree relative pos**	119 (18.1%)	93 (19.1%)	26 (15.3%)		3 (15.0%)	4 (15.4%)
**First degree relative neg**	539 (81.9%)	395 (81%)	144 (84.7%)		17 (85.0%)	22 (84.6%)
**Total**	658	488	170	p = 0.27	20	26
**Pre/peri-menopausal**	177 (28.8%)	130 (28.6%)	47 (29.2%)		6 (31.6%)	8 (32%)
**Post-menopausal**	438 (71.2%)	324 (71.4%)	114 (70.8%)		13 (68.4%)	17 (68%)
**Total**	615	454	161	p = 0.89	19	25
**Tumour grade 1**	135 (21.8%)	112 (25%)	23 (14.6%)		0 (0%)	3 (12.0%)
**Tumour grade 2**	290 (46.8%)	224 (48.6%)	66 (41.8%)		12 (63.2%)	12 (48.0%)
**Tumour grade 3**	194 (31.3%)	125 (27.1%)	69 (43.7%)		7 (36.8%)	10 (40.0%)
**Total**	619	461	158	**p<0.001**	19	25
**Lymph node negative**	385 (63.6%)	298 (66.1%)	87 (56.5%)		5 (27.8%)	14 (53.9%)
**Lymph node positive**	220 (36.4%)	153 (33.9%)	67 (43.5%)		13 (72.2%)	12 (46.1%)
**Total**	605	451	154	**p = 0.03**	18	26
**Non Triple Negative (NTN)**	585 (89.2%)	452 (92.6%)	133 (79.2%)		19 (95.0%)	22 (95.7%)
**Triple Negative (TN)**	71 (10.8%)	36 (7.4%)	35 (20.8%)		1 (5.0%)	1 (4.3%)
**Total**	656	488	168	**p<0.001**	20	23

Numbers for each sample group are given in each column. Cases with blood only are compared to cases with blood and tumour available.

### Methylation levels vary between CpG sites, and are higher in tumour DNA compared to blood DNA

**Fig 2:**
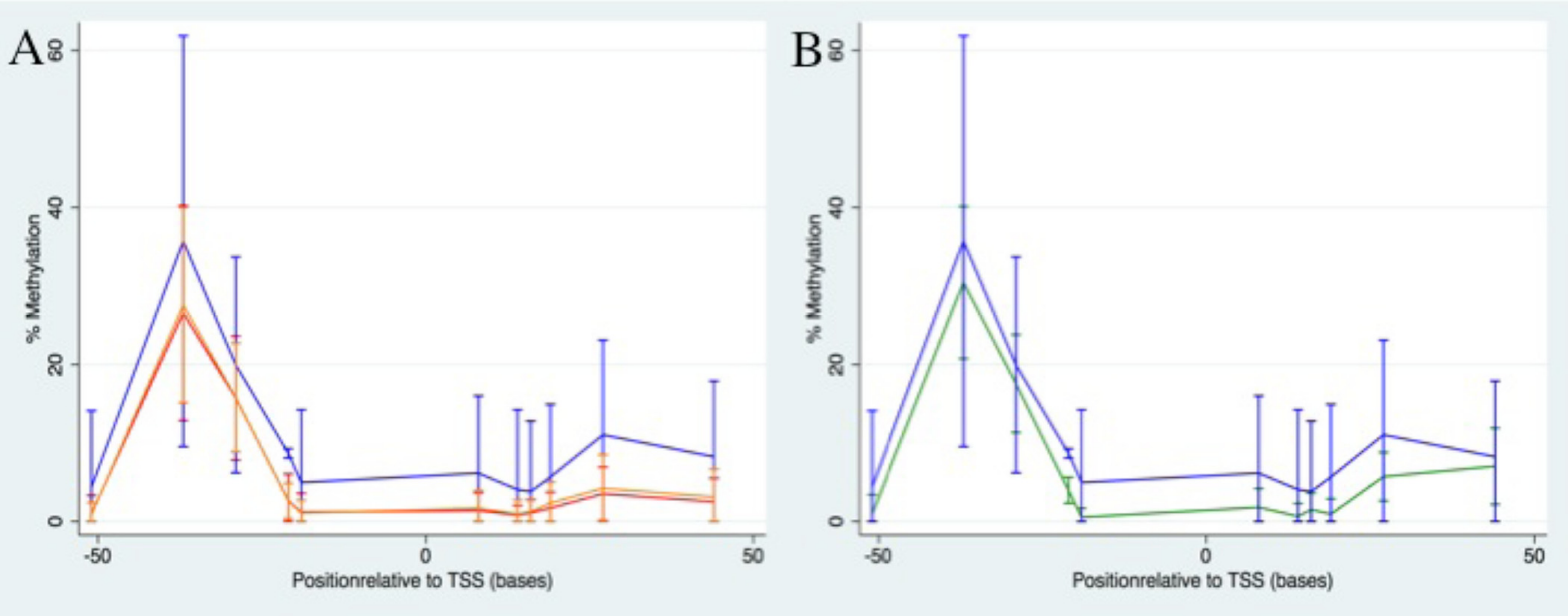
Methylation plots comparing blood, tumour and normal breast tissue. Mean (+/-SD) methylation levels plotted against CpG site position along the chromosome in relation to the *BRCA1* transcription start site (position zero). (A) Blood DNA methylation level is shown in red and tumour DNA methylation level in blue for the matched samples (n = 170) and blood DNA methylation level for the whole sample set is shown in orange (n = 658). (B) Normal breast tissue DNA methylation level is shown in green (n = 26) and tumour DNA methylation level in blue (n = 170).

The levels of *BRCA1* promoter methylation at 9 of the 11 CpG sites in blood DNA were found to correlate with methylation at the corresponding sites in matched tumour DNA in the 170 cases for which both were available. Specifically, methylation levels at all sites apart from +27 and +44 were significantly correlated at p<0.0007 (Fig 2A and S2 Table). The levels of methylation were significantly higher in tumour DNA compared to matched blood DNA at all sites apart from +16 (p<0.0025; Fig 2A and S2 Table). Blood DNA methylation levels for subjects with matched tumour (n = 170) were representative of the larger set of blood DNA methylation data (n = 658; Fig 2A). Furthermore, in both blood and tumour DNA there was a distinctive pattern of methylation around the transcription start site, with higher levels of methylation at -37 and -29 compared to the other sites (Fig 2A). DNA was available from normal breast tissue for 26 cases. The *BRCA1* promoter methylation levels were generally higher in tumour DNA compared to normal breast tissue DNA, most significantly at -51, -21, -19 and +19 (p<0.03; Fig 2B and S2 Table). For 20 cases, two tumour FFPE blocks from the same tumour were available. The methylation levels were significantly correlated between the pairs of blocks for all CpG sites except -51 and +44 (p<0.04; S3 Table).

### *BRCA1*-like morphological features are associated with triple negativity, loss of BRCA1 protein expression and higher grade

**Fig 3:**
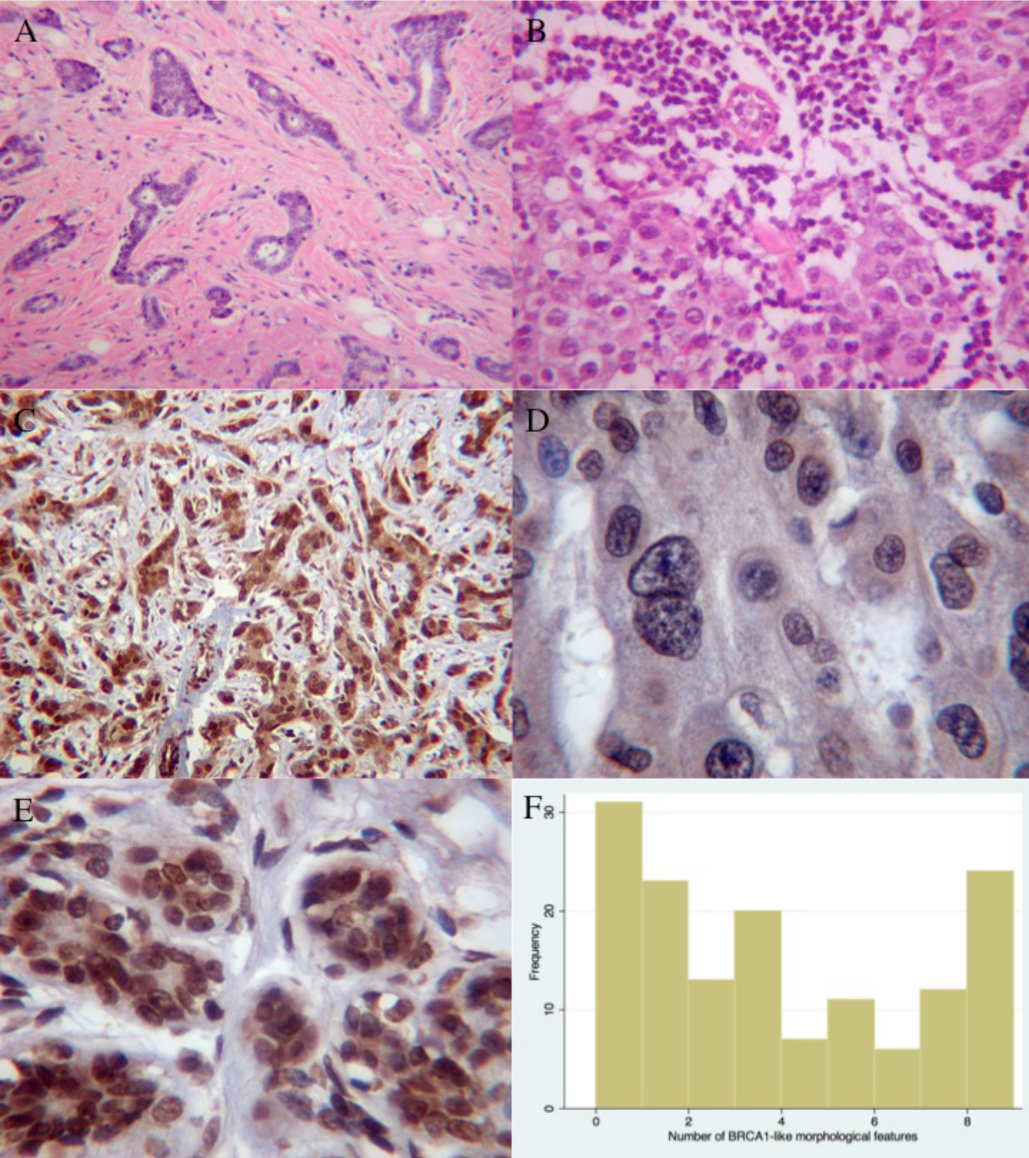
Morphological features and BRCA1 expression. H&Es demonstrating BRCA1 associated morphological features scores. A: Low score demonstrating good tubule formation, little nuclear pleomorphism, no lymphocytes and no mitoses. B: High score demonstrating syncytial islands, marked nuclear pleomorphism and heavy lymphocytic infiltrate. C, D&E: IHC performed using BRCA1 specific antibody Ab-1. C: Breast tumour demonstrating nuclear staining for BRCA1. D: Breast tumour demonstrating lack of nuclear or cytoplasmic staining for BRCA1. E: Normal breast tissue demonstrating normal nuclear staining for BRCA1. F: Histogram of the number of BRCA1-like morphological features from zero to 9 (n = 147).

We were able to score 147 tumours for the presence of 9 BRCA1-like morphological features (Fig 3), and tumours were then grouped according to whether they exhibited five or more features [33]. BRCA1 protein expression was assessed by immunohistochemistry in 119 cases (Fig 3). Higher levels of BRCA1 protein expression were associated with fewer BRCA1-like morphological features (p = 0.019; Table 3). The number of morphological features was strongly associated with triple negativity, larger tumour size and higher grade (p<0.0001, p = 0.034, p<0.0001 respectively; Table 3).

**Table 3:** Morphological feature scoring.

Number of morphological features (n)	Mean age in years	Median grade	Mean size (mm)	Number TN (%)	BRCA1 expression (%positive)
≥5 (53)	57.0	2.8	25.4	26 (76.5)	9 (23.7)
<5 (94)	58.8	2.1	20.7	8 (23.5)	29 (76.3)
	p = 0.41	**p<0.0001**	**p = 0.034**	**p<0.0001**	**p = 0.019**

Comparison of number of BRCA1-like morphological features with clinicopathological features; age, grade, size, TN receptor status and BRCA1 expression.

### Levels of both blood and tumour DNA methylation are higher in tumours with high BRCA1-like features scores

**Fig 4:**
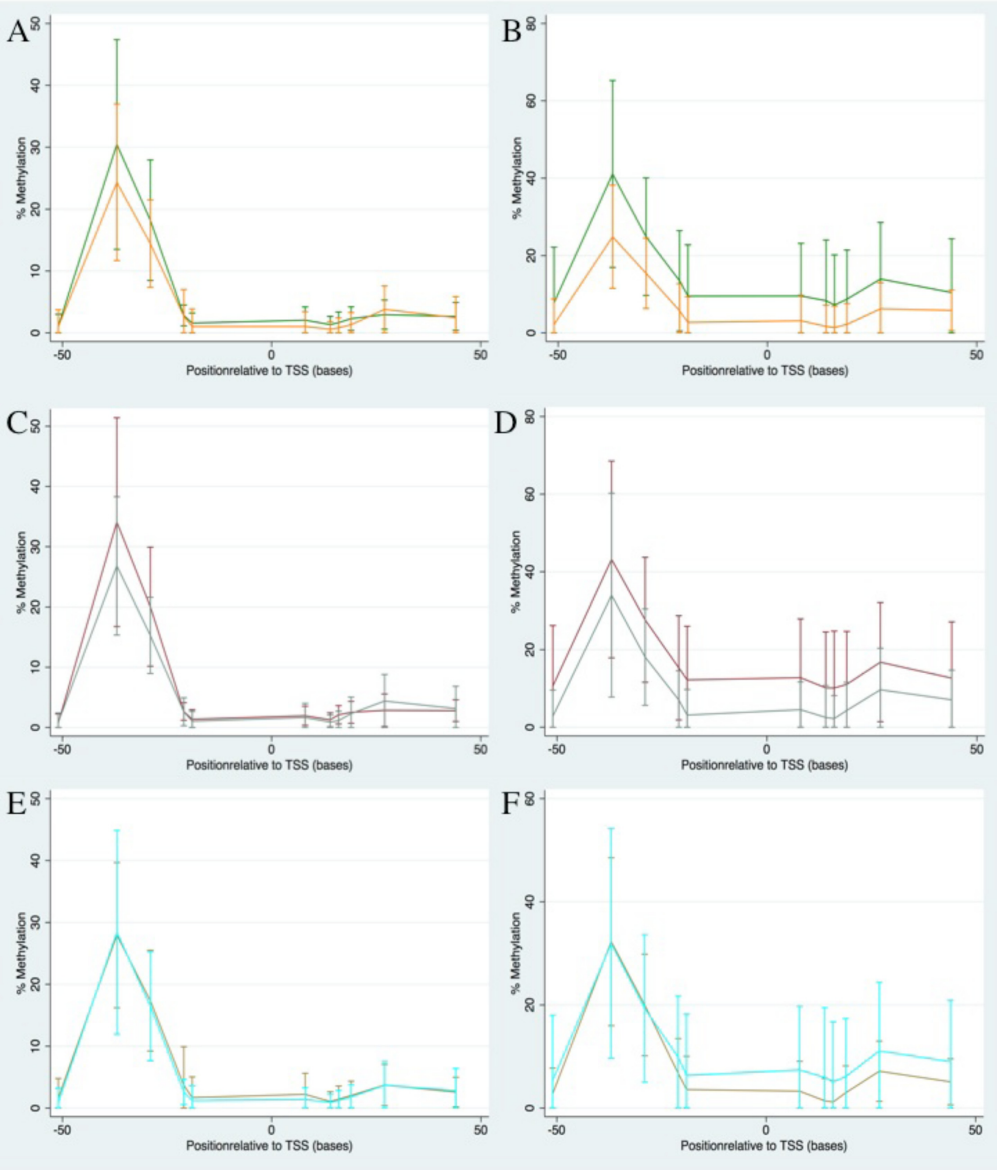
Methylation plots comparing blood and tumour for BRCA1-like features, Triple negativity and BRCA1 protein expression. Mean (+/-SD) methylation levels plotted against CpG site position along the chromosome in relation to the *BRCA1* transcription start site (position zero). Blood methylation level is shown on the left and tumour methylation level on the right. A&B: Tumours with <5 BRCA1-like features are shown in orange and those with ≥5 BRCA1-like features are shown in green. C&D: Triple negative tumours are shown in maroon and non-triple negative are shown in teal. E&F: Tumours with high levels of BRCA1 protein expression are shown in brown and those with low levels are shown in cyan.

The levels of tumour methylation were significantly higher in cases with tumours having more than or equal to 5 BRCA1-like features compared to those with less than 5, at all CpG sites except +44 (p = 0.007 to p<0.0001; Fig 4A and 4B and S4 Table). This pattern was also seen at the majority of CpG sites in blood DNA, although the differences were less statistically significant. Interestingly, the +27 CpG site was an exception, where the levels of blood methylation were not significantly different in the group with over 5 BRCA1-like features compared to those with fewer than 5 (p = 0.08 in blood, p<0.0001 in tumour; S4 Table)

### Levels of both blood and tumour DNA methylation are higher in triple negative tumours

Tumour DNA methylation was available for 35 triple-negative cases and 133 non-triple negative cases, and blood DNA methylation was available for 71 triple-negative and 585 non-triple negative cases. The levels of both blood and tumour methylation were generally higher in triple negative tumours compared to non-triple negative, at the majority of CpG sites apart from -51, -29, +19 and +44 (p value range <0.0001 to 0.03 for blood DNA, and 0.0001 to 0.01 for tumour DNA; Fig 4C and 4D and S5 Table). Again the +27 CpG site was the exception, with lower methylation levels in blood DNA of TN cases compared to non-triple negative cases, in contrast to the higher levels in tumour DNA from TN cases (p = 0.0008 in blood and p = 0.0003 in tumour; S5 Table).

### Levels of tumour and blood methylation compared to BRCA1 protein expression levels

There were 38 cases with high BRCA1 protein expression and 81 with low expression for whom blood and tumour DNA methylation were available. There was no difference in blood methylation levels at any CpG site between those with high or low levels of BRCA1 expression. The levels of tumour DNA methylation were generally higher in the samples with lower levels of BRCA1 protein although the differences were not statistically significant (Fig 4E and 4F; S6 Table).

## Discussion

### *BRCA1* promoter methylation may be used to guide therapy

Tumours arising in women with hereditary *BRCA1* mutations tend to be TN and basal-like, features that are associated with a poor prognosis. However, in recent years it has become possible to exploit the DNA-repair defects in tumours carrying *BRCA1* or *BRCA2* gene mutations using PARP inhibitors [37, 38]. The response of *BRCA1* mutation-associated cancers to both PARP inhibitors and cisplatin-based chemotherapeutic agents has driven the interest in identifying tumours with a similar DNA-repair deficient phenotype, so that these difficult to treat patients might also benefit from targeted therapies. DNA methylation of the *BRCA1* promoter is a moderately frequent event in sporadic breast tumours and an alternative mechanism of *BRCA1* inactivation. *In vitro* studies suggest that cells with *BRCA1* CpG island methylation are also sensitive to PARP1 inhibitors and tumour *BRCA1* promoter methylation predicts response to platinum based chemotherapy agents and PARP inhibitors [32, 39, 40].

A recent meta-analysis of *BRCA1* promoter methylation studies reported an association between *BRCA1* methylation and BRCA-like clinico-pathological features such as lymph node metastasis, histological grade 3, ER and PR negativity, triple-negative phenotype and decreased BRCA1 protein expression [41]. However the majority of studies included in the meta-analysis used methylation-specific PCR or other methods that do not distinguish individual CpG sites, limiting mechanistic interpretation. Even a recent study of *BRCA1* promoter methylation using pyrosequencing analysed the results by averaging the methylation levels across all sites, thus not utilizing the CpG site-specific results generated by pyrosequencing [42]. The considerable heterogeneity between studies highlights the difficulties in drawing meaningful conclusions when different CpG sites have been studied, methylation detection methods used, populations studied and tissues examined [40]. In this study we have used pyrosequencing to distinguish methylation levels at individual CpG sites in the *BRCA1* promoter, and analysed CpG sites that had been studied in at least three previous studies [23, 43, 44]. We have focused on obtaining a comprehensive dataset consisting of methylation levels for blood and tumour DNA, BRCA1 protein expression, hormone receptor status, and morphological and clinico-pathological features.

### Blood and tumour methylation levels are related to BRCA-like phenotypes

We found that there was a strong correlation between methylation levels in blood and tumour DNA at all CpG sites apart from +27 and +44, with levels in the tumour being consistently higher than those in the blood (or normal breast tissue), at all CpG sites. Whether the strong correlation between these different tissues reflects independent events, constitutional changes or global methylation changes secondary to carcinogenesis is beyond the scope of this study due to its retrospective nature; prospective analyses are required to determine whether blood methylation could be used as a predictor of tumour methylation [45, 46]. The effect of DNA methylation on gene expression is complex with both hypo- and hypermethylation at specific gene regions differentially affecting gene expression [47], however there is limited mechanistic work on individual CpG sites.

Consistent with previous observations, we found that BRCA1-like morphological features are correlated with triple negativity, loss of BRCA1 protein expression and higher grade. The levels of both blood and tumour DNA methylation at most CpG sites were higher in tumours with high BRCA1-like features scores and were also higher in triple negative tumours, as was shown by Wong et al [23]. The overall picture was that the associations in blood DNA were weaker than those in tumour DNA but in the same direction. The +27 CpG site was an exception to this general pattern where the associations tended to be in the opposite direction in blood and tumour DNA. We had limited power to detect associations with BRCA1 protein expression, and although mean tumour DNA methylation (at sites +8 to +44 in particular) were higher in tumours with lower levels of BRCA1 protein, these effects did not reach statistical significance. Previous studies have shown a relationship between DNA methylation and BRCA1 protein [24, 48, 49].

Other methylation analysis techniques more commonly used, including MSP (Methylation Specific PCR), MS-HRM (methylation-sensitive high-resolution melting) Methyl-light and MS-MLPA (methylation-specific multiplex ligation-dependent probe amplification) do not give as much detailed information about the methylation status and often quintiles or arbitrary cut off points are chosen to define ‘methylated’ or ‘unmethylated’ promoters. The meta-analysis by Zhang et al found that over half of the studies included used MSP as their predominant method for analysing methylation, whilst only one study used pyrosequencing [41, 50]. Methylation analysis using pyrosequencing is becoming widely used in diagnostics laboratories, which may drive further translational research [51]. Future studies may need to focus on appropriate methods of methylation analysis to detect levels in biopsy specimens, particularly in TN tumours, because of the increasing use of neo-adjuvant chemotherapy.

The distribution of histopathological features, including receptor status, age at diagnosis and grade, can be used to predict women more likely to harbour germline *BRCA1* or *BRCA2* mutations [52]. Scoring breast tumours for morphological features associated with ‘BRCAness’, as has been performed in our study, can be used to help identify which tumours may have higher levels of promoter methylation [23, 33]. This information could be used in future studies alongside receptor status, age at diagnosis and histological grade to select a subgroup of patients for epigenetic and genetic testing and subsequent targeted therapies. The use of tumour histopathology is gaining acceptance as a way to target costly and time-consuming genetic testing to ‘at-risk’ individuals based on their tumour characteristics [53].

This study highlights the variability in methylation level at different CpG sites close to the BRCA1 transcription start site. Methylation levels in tumour are generally greater than those in blood, and methylation at most sites (apart from +27) increases in triple negative tumours and those with a high BRCA1-like features scores. Analysis of *BRCA1* promoter methylation may contribute to strategies for the identification of women who may benefit from PARP inhibition or other targeted therapies, as has occurred in BRCA associated ovarian cancer [54].

## Supporting Information

S1 Table Data set used for analysis. (XLSX)

S2 Table Summary table comparing blood and tumour methylation at individual sites for matched tumour and blood samples, the full cohort of blood samples and matched normal and tumour tissue. The paired sign-rank tests have been used.
(XLSX)

S3 Table Correlation of methylation levels between paired FFPE blocks from the same tumour determined using Spearman’s rho. (XLSX)

S4 Table Comparison between blood and tumour methylation based on scoring of *BRCA1* associated features. (XLSX)

S5 Table Comparison between blood and tumour methylation based on Triple Negative (TN) or Non-Triple Negative (NTN) receptor status. (XLSX)

S6 Table Comparison between blood and tumour methylation based on the presence or absence of BRCA1 expression determined by immunohistochemistry (IHC). (XLSX)
